# Xerogenic medications may contribute to decreased unstimulated salivary flow in patients with oral burning and/or gastro-esophageal reflux disease

**DOI:** 10.3389/fdmed.2023.1047235

**Published:** 2023-02-23

**Authors:** Linda Sangalli, Walied Eldomiaty, Craig S. Miller

**Affiliations:** ^1^ Department of Oral Health Science, Division of Orofacial Pain, College of Dentistry, University of Kentucky, Lexington, KY, United States; ^2^ College of Dental Medicine - Illinois, Midwestern University, Downers Grove, IL, United States; ^3^ Periodontics Division, College of Dentistry, University of Kentucky, Lexington, KY, United States; ^4^ Chief, Periodontics Division, College of Dentistry, University of Kentucky, Lexington, KY, United States

**Keywords:** adverse effects, burning mouth syndrome, gastroesophageal reflux disease, glossodynia, xerostomia

## Abstract

**Background:**

Patients who complain of mucosal burning sensations (i.e., glossodynia and gastro-esophageal reflux disease, GERD) often take multiple medications. However, the relationship between xerogenic medication intake and salivary flow in these patients has not been thoroughly examined.

**Methods:**

A retrospective study of 192 consecutive patients diagnosed with glossodynia (ICD-10-CM) at a regional center over a six-year period was performed. Data from electronic health records were extracted and relationships between medication intake, unstimulated whole salivary flow rate (UWSFR), xerostomia, and GERD were determined by chi-square, *t*-test, and correlation analysis.

**Results:**

Of 134 records that met inclusion criteria, 87.1% of patients reported daily intake of one or more xerogenic medications. Two or more xerogenic medications were taken significantly more often by patients with glossodynia reporting GERD than those with glossodynia without GERD (*p *= .02). UWSFR was negatively correlated with number of medications [*r*(103) = −.277, *p *= .005] and xerogenic medications [*r*(103) = −.195, *p *= .049]. The lowest UWSFR was observed with use of trazodone and cyclobenzaprine.

**Conclusions:**

Daily xerogenic medication intake, hyposalivation, and xerostomia were commonly present and potentially interrelated in patients who suffer from glossodynia and/or GERD.

**Practical implications:**

Clinicians should be aware of the consequences of prescribing multiple and certain xerogenic medications in reducing UWSFR, especially in patients physiologically at risk of hyposalivation such as those suffering from GERD and/or glossodynia.

## 1. Introduction

Saliva is a complex fluid comprised of major and minor salivary gland secretions important for intraoral hard and soft tissues (1), neutralizing acids in the oral cavity (2), and decreasing the time that acid is in contact with esophageal mucosa (3). A healthy individual produces on average 500 ml–1.5 L of saliva daily, about 0.35 ml/min (4).

Alterations in saliva, quality or quantity, can result from several etiological factors, including systemic conditions (5), such as obesity (6), diabetes (7), autoimmune disease (Sjogren's syndrome) (8), syndromes (Down syndrome) (9), neurodegenerative diseases (Alzheimer's disease) (10), infections [hepatitis (11), HIV-infection (12)]; iatrogenic cause, such as hemodialysis (13), radioactive iodine therapy (14), radio- or chemotherapy (15, 16); environmental factors, like dietary change, tobacco, alcohol or recreational drug use (17); physiological changes (18), including aging (19, 20); or medication intake (21–24). Medications, alone or in combination, are routinely prescribed to treat systemic or local conditions. However, many medications induce dry mouth and low salivary flow (25), which can contribute to mucosal atrophy and fissuring (26), dental caries (27), and fungal infections (28).

Reduced salivary volume may also contribute to glossodynia. Among studies that investigated this association, on average 64% of patients with glossodynia have xerostomia (range 23%–100%) (29–38) and 56% have hyposalivation (range 33%–100%) (29, 31–34, 37–44). Despite the established association between glossodynia and diminished salivary flow, to the best of our knowledge their relationship with xerogenic medications has not been thoroughly examined.

A comorbidity frequently seen among patients with glossodynia is gastro-esophageal reflux disease (GERD), a condition known to produce burning sensation. This can be initiated or maintained from a reduced salivary volume, which may itself contribute to symptoms felt in anatomical structures distal to the mouth (45). In the available literature, eleven studies measured unstimulated salivary flow (UWS) in patients with GERD, and four of these conducted on a total of 351 subjects confirmed a significant reduction in UWS, compared to 297 healthy controls (46–49). Moreover, patients with GERD tend to be managed with further medication intake, that can contribute to decreased salivary volume. Hence, the vicious cycle between xerogenic medications, salivary flow, and GERD may be important. However, few studies have investigated the presence of GERD in patients diagnosed with glossodynia with respect to xerogenic medications (50–55).

Therefore, the objectives of this study were to investigate the patients diagnosed with glossodynia (1) the prevalence of xerogenic medication; (2) the association between salivary flow volume and medication use; (3) which medications were associated with hyposalivation; and (4) the presence and association of xerostomia and hyposalivation with or without GERD. The null hypothesis was that patients with glossodynia are unlikely to take xerogenic medications and have concurrent GERD symptomatology.

## 2. Methods

A retrospective study was conducted on data collected from consecutive patients with a complaint of burning mouth sensation, seen at the Orofacial Pain Center (University of Kentucky, Lexington, United States) between January 2014 and April 2020. Due to the retrospective design of the study, a sample size calculation was not performed. However, a post-hoc power analysis on a sample size of 103 revealed a power of 81%.

Patient's electronic health records were reviewed for eligibility, according to the following inclusion criteria: (1) diagnosis of glossodynia (International Classification of Disease ICD-10-CM, Diagnosis Code-14.6); and (2) oral burning pain rated greater than 0, on a 0–10 numerical rating scale (NRS, 0 = “no pain” and 10 = “worst possible pain”). Patients were categorized as presenting with GERD symptomatology when they had either a physician diagnosis or self-reported GERD on intake forms. Exclusion criteria included patients with complaints of oral burning due to known condition (anemia, oral lesion, diabetes mellitus, Sjögren's syndrome, vitamin deficiency, or use of angiotensin converting enzyme inhibitors). Although GERD has been classified among underlying medical conditions associated with oral burning symptoms, for the current investigation, participants with a complaint of GERD were included in the study population. The study was approved by the Institutional Review Board of the Office of Research Integrity at the University of Kentucky (Lexington KY, United States; IRB #74332).

### 2.1. Procedure

All patients underwent a clinical evaluation for their oral burning complaint by the same Diplomate of the American Board of Oral Medicine (C.M.). A thorough medical history was collected with specific questions that investigated chief complaint, systemic medical conditions, medications, parafunctional habits, alcohol consumption, smoking status, daily physical activity, and laboratory results.

#### 2.1.1. Oral burning sensation

The chief complaint was investigated by ascertaining the pain location, quality, frequency, and duration, the presence of taste disturbances, and triggering, aggravating, and relieving factors. Pain intensity was collected on a 0–10 NRS, with 10 being the worst possible pain.

#### 2.1.2. Medications

The number and types of prescribed medication were recorded, with a focus on those known to be xerogenic (21, 22, 56).

#### 2.1.3. Xerostomia

Xerostomia, defined as a subjective complaint of dry mouth (57–59), was assessed by self-report or with the Short-Form Xerostomia Inventory questionnaire (SXI) (60). The following statements on the SXI: “My mouth feels dry”; “My mouth feels dry when eating a meal”; “I have difficulties in eating dry food”; “I have difficulties swallowing certain foods”; and “My lips feel dry” were scored as “Never” 1; “Occasionally” 2; and “Often” 3. A cut-off of 10 or higher was used as clinical definition of “xerostomia” (personal communication with WM Thomson) (60).

#### 2.1.4. Hyposalivation

Unstimulated whole salivary flow rate (UWSFR) was measured by instructing the patient to allow saliva to collect in the mouth and spit into a cup every 20 s for 5 min, while sitting upright and undisturbed in a comfortable position. Samples were collected between 1 pm and 4 pm, with participants refraining from eating or drinking one hour before the procedure (61). Hyposalivation was defined as UWSFR < 0.2 ml/min, which represents the lowest 10th percentile as determined in our clinic (data not shown) and many previous studies (62, 63).

UWSFR of all patients was measured by the same Diplomate of the American Board of Oral Medicine (C.M.) to increase the reproducibility and standardization of the procedure.

### 2.2. Statistical analysis

Data normality was checked with a Shapiro-Wilk test. Variables were expressed as means and standard deviations. Hyposalivation, GERD symptomatology, and xerostomia were coded as dichotomous variables (1 = yes, 0 = no). Medications and salivary flow rate were coded as continuous variables. Descriptive statistics were completed for patients with data on salivary flow rate and xerostomia. Bivariate correlation analysis was performed to test the association between UWSFR and medication (number of medications, number and types of xerogenic medications), and between UWSFR and GERD. According to GERD symptomatology, the total population was divided in two groups (patients presenting with GERD symptoms = 1; patients not presenting with GERD symptoms = 0). Chi-square and McNemar tests were used when appropriate to compare the two groups in terms of gender, number, and type of medication. Independent *t*-test was used to compare the two groups in terms of age, medical conditions, pain intensity, xerostomia, and UWSFR. Statistical significance was set at *α* = 0.05. Data were analyzed with SPSS (IBM SPSS Statistics Macintosh, Version 27.000, IBM Corp, Armonk, NY).

## 3. Results

Of 192 records identified, 58 were excluded due to missing data or a diagnosis different from glossodynia (IDC-10 14.6). A total of 134 patients diagnosed with glossodynia (mean age of 63.43 ± 11.84, 80.6% females) were included in the analysis (Table 1). The mean pain intensity was 6.30 ± 2.39, and 38.1% reported a taste disturbance. On average, patients had 5.17 ± 3.28 systemic medical conditions, with hypertension being the most common (44.8%), followed by GERD (44.0%) and depression (22.4%). Salivary flow was measured in 104 patients (77.6%). Their mean UWSFR was 0.24 ± 0.30 ml/min, and 68 (65.4%) had hyposalivation. Xerostomia was determined by questionnaire to be present in 76 patients out of 98 (77.6%), and the SXI yielded a mean score of 12.84 ± 5.90. Seventy-eight percent of xerogenic patients had hyposalivation [*χ*^2^(104) = 15.11, *p *= .001]. Of those, 38 (50.7%) reported GERD symptoms.

**Table 1:** Characteristics of the study population and differences between participants with and without GERD symptomatology.

	Total (*N* = 134)	GERD symptomatology (*N* = 59)	No GERD symptomatology (*N* = 75)	*p* value*
**Gender (%)**				
Male	26 (19.40)	13 (22.03)	13 (17.33)	.517
Female	108 (80.60)	46 (77.97)	62 (82.67)	
Age (years), mean ± SD	63.43 ± 11.84	62.79 ± 12.11	63.94 ± 11.68	.579
N medical conditions, mean ± SD	5.17 ± 3.28	6.64 ± 3.83	4.03 ± 2.21	.000
Taste disturbance (%)	51 (38.06)^¥^	20 (33.90)	31 (41.33)	.381
Pain intensity, mean ± SD	6.30 ± 2.39^§^	6.09 ± 2.56	6.46 ± 2.25	.411
Reported dry mouth (%)	76 (77.55)^£^	38 (82.61)	38 (73.08)	.192
UWSFR, mean ± SD	0.24 ± 0.30^†^	0.23 ± 0.27	0.24 ± 0.32	.980

GERD: gastro-esophageal reflux disease; UWSFR: Unstimulated Whole Salivary Flow Rate.

¥Value calculated on a total of 124 subjects.

§Value calculated on a total of 121 subjects.

£Value calculated on a total of 98 subjects.

†Value calculated on a total of 104 subjects.

*p-values obtained from a chi-square test for categorical variables, and from an independent sample *t*-test for continuous variables.

### 3.1. Medication and salivary flow rate

**Figure 1:**
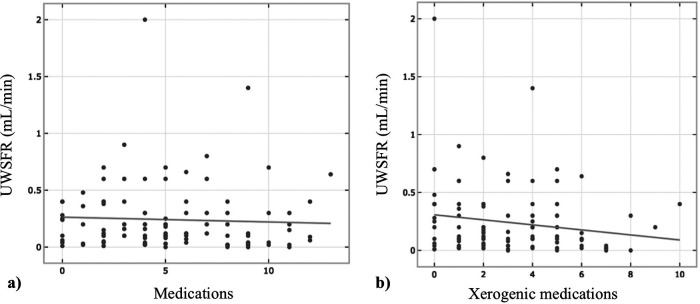
Relationship between number of medications (**A**) and xerogenic medications (**B**) and unstimulated whole salivary flow rate (UWSFR) (*N* = 104).

The mean number of daily prescribed medications for the 134 patients diagnosed with glossodynia was 5.5 ± 3.49. A significant negative correlation was found between the number of medications and UWSFR [*r*(103) = −.277, *p *= .005, Figure 1A]. The mean number of daily xerogenic medications of the total sample size was 3.11 ± 2.22. Overall, 72.7% reported taking 2 or more xerogenic medications; 14.4% reported taking 1 xerogenic medication, and 12.9% of the total participants did not report any daily xerogenic medication intake. A negative correlation was found between the number of xerogenic medications and UWSFR [*r*(103) = −.195, *p *= .049, Figure 1B]. The most common xerogenic medications were selective serotonin reuptake inhibitors (SSRIs, 31.3%) and non steroid anti-inflammatory drugs (NSAIDS, 13.4%) (Table 2). Daily use of omeprazole was ascertained in 27.6% of the patients. Daily cyclobenzaprine [*r*(104) = −.201, *p *= .041] and ibuprofen use [*r*(104) = −.206, *p *= .036] were negatively associated with UWSFR. The medication profiles between patients reporting GERD and those not reporting GERD were similar (all *p*'s > 0.05, Table 2). Not surprisingly, daily intake of omeprazole was significantly higher in those with GERD symptoms (39.0% vs. 18.7%, *p *= .009). Also, two or more xerogenic medications were used significantly more often by patients with glossodynia reporting GERD than those who took fewer than two xerogenic meds [*χ*^2^(132) = 7.85, *p *= .020].

**Table 2:** Number and type of medication intake in the study population and differences between participants with and without GERD symptomatology.

	Total (*N* = 134)	GERD symptomatology (*N* = 59)	No GERD symptomatology (*N* = 75)	*p* value*
Mean number of medications ± SD	5.5 ± 3.49	6.12 ± 3.46	5.02 ± 3.46	.074
Mean number of xerogenic medications ± SD	3.11 ± 2.22	2.02 ± 2.03	2.35 ± 0.27	.157
SSRIs (%)^a^	42 (31.34)	18 (30.51)	24 (32.00)	.853
Paroxetine	2 (1.49)	1 (1.69)	1 (1.33)	1.000
Escitalopram	14 (10.45)	7 (11.86)	7 (9.33)	.634
Alprazolam	11 (8.21)	2 (3.39)	9 (12.00)	.111
Trazodone	7 (5.22)	1 (1.69)	6 (8.00	.134
Sertraline	4 (2.99)	2 (3.39)	2 (2.67)	1.000
Fluoxetine	3 (2.24)	1 (1.69)	2 (2.67)	1.000
Buproprion	12 (8.96)	7 (11.86)	5 (6.67)	.296
**PPI**				
Omeprazole	37 (27.61)	23 (38.98)	14 (18.67)	.009*
**Muscle relaxant**				
Cyclobenzaprine	6 (4.48)	2 (3.39)	4 (5.33)	.694
NSAIDS	18 (13.43)	9 (15.25)	8 (10.67)	
Ibuprofen	7 (5.22)	5 (8.47)	2 (2.67)	.240
Meloxicam	6 (4.48)	2 (3.39)	4 (5.33)	.694
Naproxen	5 (3.73)	3 (5.08)	2 (2.67)	.654
ACE				
Lisinopril	17 (12.69)	7 (11.86)	10 (13.33)	.506
**Calcium Channel Blocker**				
Amlodipine	15 (11.19)	5 (8.47)	10 (13.33)	.376
**Sedative-hypnotics**				
Zolpidem	14 (10.45)	7 (11.86)	7 (9.33)	.634
SNRIs	11 (8.21)	5 (8.47)	6 (8.00)	.921
Duloxetine	8 (5.97)	4 (6.78)	4 (5.33)	.731
Venlafaxine	3 (2.24)	1 (1.69)	2 (2.67)	.717

ACE, Angiotensin-Converting Enzyme; *N*, number; NSAIDS, nonsteroidal anti-inflammatory drugs; PPI, Proton Pump Inhibitors; SD, standard deviation; SNRIs, Serotonin Norepinephrine Reuptake Inhibitors; SSRIs, Selective Serotonin Reuptake Inhibitors.

^a^ Some patients took more than one SSRI.

*p-values were obtained from a chi-square test or McNemar test for categorical variables as appropriate, and from an independent sample *t*-test for continuous variables.

**Figure 2:**
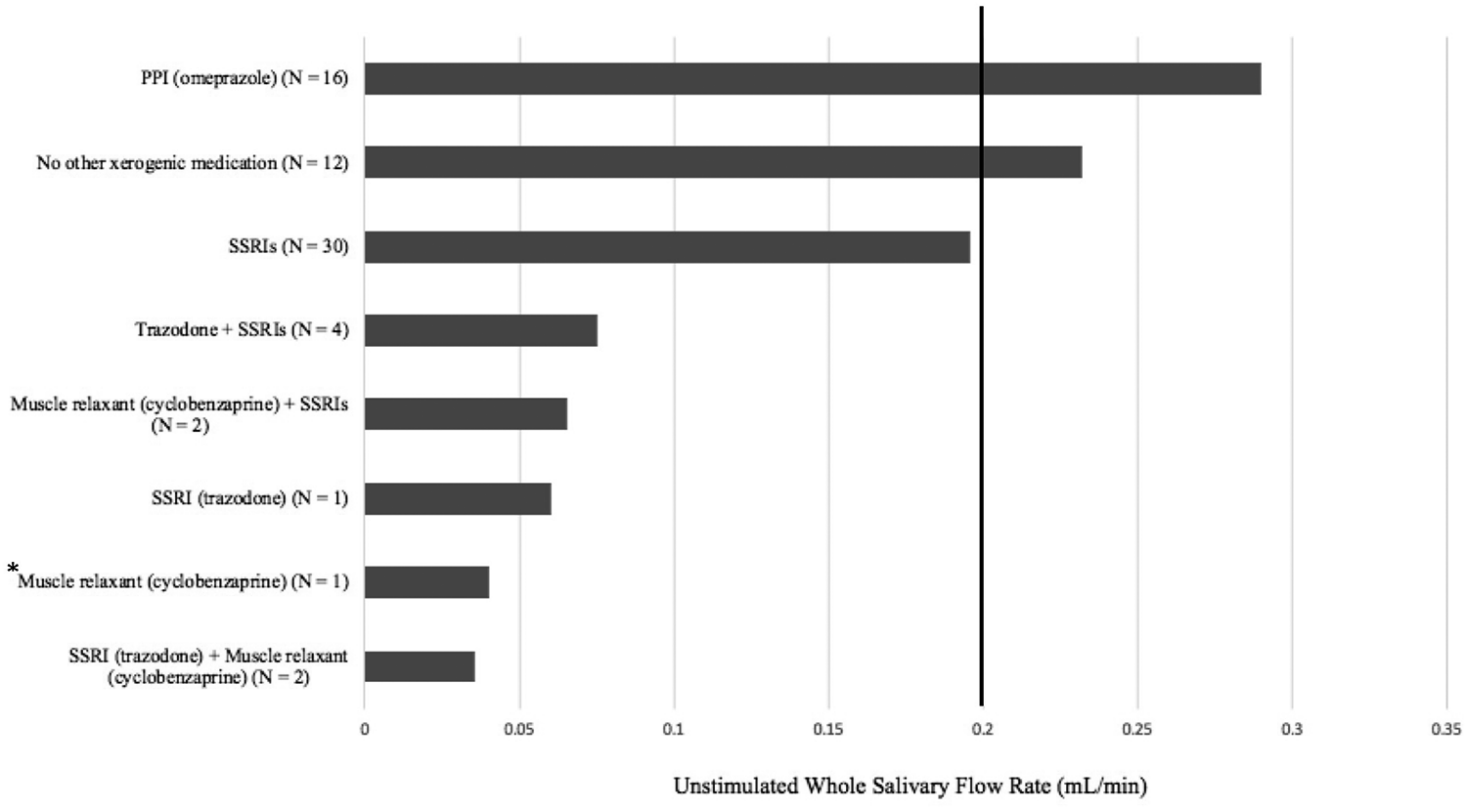
Type of medication and unstimulated whole salivary flow rate (UWSFR).

Figure 2 displays the overall mean UWSFR of patients who used xerogenic medications daily. Those with daily intake of trazodone and cyclobenzaprine alone or in combination with another drug had the lowest UWSFR (0.03 ± 0.02 ml/min); those using omeprazole did not present with hyposalivation (0.29 ± 0.19 ml/min).

### 3.2. UWSFR, xerostomia, and GERD

Fifty-nine of those with glossodynia (44.0%) reported GERD. Of those, 61.4% had hyposalivation. A significant difference was not found in UWSFR between patients who reported GERD symptoms and those who did not (Table 2). A difference in xerostomia was not found between participants with and without GERD [*χ*^2^(134) = 2.540, *p *= .194].

## 4. Discussion

This study is one of the first to explore the interaction between medication intake and salivary flow in patients with glossodynia, with or without GERD symptomatology. The findings indicate that the majority of patients complaining of intraoral burning took xerogenic medications. A positive association was found between the number of xerogenic medications and low UWSFR in those with glossodynia. A high prevalence of GERD symptomatology (44%) was observed in patients with oral burning sensations and daily xerogenic medication intake. Despite this frequency, the presence of GERD and omeprazole use by themselves were not related to salivary flow rate.

Xerostomia was identified in 78% of patients diagnosed with glossodynia in this study, which is higher than the prevalence of xerostomia found in population-based studies (ranges from 0.9% to 64.8%) (64–67). Several factors may explain the greater prevalence of xerostomia in our patient population. In our clinic, the intake forms ask specifically about xerostomia whereas other studies may not. Also, xerostomia is common with aging and with use of xerogenic medications. Many of our patients took xerogenic medications, and many other studies did not explore the relationship with xerogenic medications (29, 31, 35, 40, 68). Finally, xerostomia is a subjective complaint based on self-reported data that may not be reliable; however, our study attempted to make the condition more objective by the administration of, and use of data from, the SXI form.

Our study demonstrated that 78% of those reporting xerostomia had hyposalivation. The present study utilized a cut-off of 0.2 ml/min to define hyposalivation, because patients below this threshold are in the lowest 10th percentile and often have symptoms or clinical findings consistent with the presence of hyposalivation-associated disorders. This is in contrast to the literature, where a cut-off of 0.1 ml/min is a commonly used definition for hyposalivation, as originally indicated by Ericsson and Hardwick (69). In the current study, hyposalivation was observed in 65% of patients diagnosed with glossodynia, which may be attributed to the age of our patient population and the extensive list of xerogenic medications used in this cohort. Alternatively, this finding may point to the potentially intrinsic common root between hyposalivation and glossodynia. In this regard, the results of the present study corroborate the negative association between number of medications and UWSFR (39, 70–77). Specifically, the lowest UWSFRs were observed in patients with glossodynia who took a daily combination of xerogenic medications, as shown in Figure 2. These observations are consistent with studies that reported difference between monotherapy and polytherapy (75, 78). However, these findings highlight a difficulty with the management of glossodynia; that is, how do clinicians who treat these patients eliminate hyposalivation when xerogenic medications are needed for management of their comorbidities? Novel study designs may be needed to address this concern to better understand confounders vs. root causes of glossodynia.

The findings from this study help support the concept that polypharmacy and hyposalivation underlie burning sensations that occur in the mouth and upper gastro-intestinal tract (53, 79–82). In this regard, 40% of those with hyposalivation also reported GERD symptomatology, although the difference was not statistically significant. Contradictory results are found in the literature, with studies supporting a relationship between hyposalivation and GERD (83–87) and studies that fail to demonstrate a correlation (53, 80, 81, 84). This may be due to the different (1) criteria used to diagnose GERD [endoscopy (53, 81, 84) vs. questionnaires and self-reported reflux symptoms (83, 47)]; and (2) assessment methods of salivary flow [different duration (80, 81, 83), frequency (53, 80, 81), and time (53, 81, 83) of spitting method (53, 80, 81, 83, 84, 85), vs. Saxon method (48, 49), salivary scintigraphy (46), or modified cotton roll method (47)].

This study has limitations. First, the retrospective design did not permit control of confounding factors, such as medical condition, dosage of the medication taken, or allow retrieval of all the data. Hence, missing items were imputed using multiple imputation as appropriate. Future studies with more rigorous methodology, which include the comparison with a healthy control group, are needed to control for factors that may influence the results. Similarly, the association observed between UWSFR and medication intake does not prove causation. Second, medication intake was self-reported and not investigated in terms of daily frequency, dosage, duration, and compliance. These factors have been shown to influence salivary flow rate (71, 86, 87). The sample included mostly elderly females, due to age and gender predilection of glossodynia. Therefore, these findings might not be generalizable to males or larger populations. Moreover, hormonal disturbances may influence the high prevalence of hyposalivation and xerostomia, which was not taken into consideration in the present investigation. As 18.7% of patient without GERD reported to take omeprazole, it is possible that they may have been misclassified. Therefore, we reanalyzed the data by classifying these patients within the GERD group, and these analyses did not alter the results (data not shown). Similarly, the presence of other comorbid systemic conditions was not included in the analysis as a confounding factor. Last, although an interesting trend was seen between UWSFR and type of medication, some of the findings are based on small sample size (e.g., medication use) and should be interpreted with caution until larger studies are performed.

## 5. Conclusions

These findings suggest that polypharmacy medication intake, hyposalivation, and xerostomia are commonly seen among patients with glossodynia and/or suffering from GERD. Because of these associations and the possible effect of burning sensation secondary to medication-induced hyposalivation, clinicians should be cautious in prescribing xerogenic medications and consider ways to deprescribe xerogenic medications in patients who display concurrent hyposalivation and mucosal burning sensations to help evaluate alleviation of symptoms.

## Data Availability

The original contributions presented in the study are included in the article; further inquiries can be directed to the corresponding author.

## References

[1] Mese H Matsuo R. Salivary secretion, taste and hypo salivation. J Oral Rehabil. (2007) 34:711–23. 10.1111/j.1365-2842.2007.01794.x 17824883

[2] Helm JF Dodds WJ Hogan WJ Soergel KH Egide MS Wood CM. Acid neutralizing capacity of human Saliva. Gastroenterology. (1982) 83:69–74. 10.1016/S0016-5085(82)80286-2 7075945

[3] Helm JF Dodds WJ Pelc LR Palmer DW Hogan WJ Teeter BC. Effect of esophageal emptying and Saliva on clearance of acid from the esophagus. New Eng J Med. (1984) 310:284–8. 10.1056/NEJM198402023100503 6690951

[4] Humphrey SP Williamson RT. A review of Saliva: normal composition, flow, and function. J Prosthet Dent. (2001) 85:162–9. 10.1067/mpr.2001.113778 11208206

[5] Saleh J Zancanaro Figueiredo MA Cherubini K Gonçalves Salum F. Salivary hypofunction: an update on aetiology, diagnosis and therapeutics. Arch Oral Biol. (2015) 60:242–55. 10.1016/j.archoralbio.2014.10.004 25463902

[6] de Andrade PAM Giovani PA Araujo DS de Souza AJ Pedroni-Pereira A Kantovitz KR Shifts in the bacterial community of Saliva give insights on the relationship between obesity and oral Microbiota in adolescents. Arch Microbiol. (2020) 202:1085–95. 10.1007/s00203-020-01817-y 32034425

[7] Khovidhunkit SO Suwantuntula T Thaweboon S Mitrirattanakul S Chomkhakhai U Khovidhunkit W. Xerostomia, hyposalivation, and oral microbiota in type 2 diabetic patients: a preliminary study. J Med Assoc Thai. (2009) 92(9):1220–8. PMID: . 19772183

[8] Jensen SB Vissink A. Salivary gland dysfunction and Xerostomia in sjögren's syndrome. Oral Maxillofac Surg Clin North Am. (2014) 26:35–53. 10.1016/j.coms.2013.09.003 24287192

[9] Kawai M Ito N Ayuse T. Changes in surface tension of Saliva in down syndrome. Eur Rev Med Pharmacol Sci. (2018) 22:6469–74. 10.26355/eurrev_201810_16060 30338816

[10] Zalewska A Klimiuk A Zięba S Wnorowska O Rusak M Waszkiewicz N Salivary gland dysfunction and salivary redox imbalance in patients with Alzheimer's disease. Sci Rep. (2021) 11:23904. 10.1038/s41598-021-03456-9 34903846 PMC8668975

[11] Maldonado JO Beach ME Wang Y Perez P Yin H Pelayo E HCV Infection alters salivary gland histology and Saliva composition. J Dent Res. (2022) 101:534–41. 10.1177/00220345211049395 35045743 PMC9052835

[12] Li Y Saxena D Chen Z Liu G Abrams WR Phelan JA HIV Infection and microbial diversity in Saliva. J Clin Microbiol. (2014) 52:1400–11. 10.1128/JCM.02954-13 24523469 PMC3993673

[13] Yu IC Liu CY Fang JT. Effects of hemodialysis treatment on Saliva flow rate and Saliva composition during in-center maintenance dialysis: a cross-sectional study. Ren Fail. (2021) 43:71–8. 10.1080/0886022X.2020.1857769 33327832 PMC7751405

[14] Singer MC Marchal F Angelos P Bernet V Boucai L Buchholzer S Salivary and lacrimal dysfunction after radioactive iodine for differentiated thyroid cancer: american head and neck society endocrine surgery section and salivary gland section joint multidisciplinary clinical consensus statement of otolaryngology, ophthalmology, nuclear medicine and endocrinology. Head Neck. (2020) 42:3446–59. 10.1002/hed.26417 32812307

[15] Arrifin A Heidari E Burke M Fenlon MR Banerjee A. The effect of radiotherapy for treatment of head and neck cancer on oral Flora and Saliva. Oral Health Prev Dent. (2018) 16:425–9. 10.3290/j.ohpd.a41364 30460355

[16] Acauan MD Zancanaro Figueiredo MA Cherubini K Gomes AP Salum FG. Radiotherapy-Induced salivary dysfunction: structural changes, pathogenetic mechanisms and therapies. Arch Oral Biol. (2015) 60:1802–10. 10.1016/j.archoralbio.2015.09.014 26454716

[17] Rad M Kakoie S Niliye Brojeni F Pourdamghan N. Effect of long-term smoking on whole-mouth salivary flow rate and oral health. J Dent Res Dent Clin Dent Prospects. (2010) 4:110–4. 10.5681/joddd.2010.028 23346336 PMC3429961

[18] Gueiros LA Soares MSM Leão JC. Impact of ageing and drug consumption on oral health. Gerodontology. (2009) 26:297–301. 10.1111/j.1741-2358.2009.00284.x 19392837

[19] Xu F Laguna L Sarkar A. Aging-Related changes in quantity and quality of Saliva: where do we stand in our understanding? J Texture Stud. (2019) 50:27–35. 10.1111/jtxs.12356 30091142

[20] Maciejczyk M Nesterowicz M Szulimowska J Zalewska A. Oxidation, glycation, and carbamylation of salivary biomolecules in healthy children, adults, and the elderly: can Saliva be used in the assessment of aging? J Inflamm Res. (2022) 15:2051–73. 10.2147/JIR.S356029 35378954 PMC8976116

[21] Arany S Kopycka-Kedzierawski DT Caprio TV Watson GE. Anticholinergic medication-related dry mouth and impacts on the salivary glands. Oral Surg Oral Med Oral Pathol Oral Radiol. (2021) 132:662–70. 10.1016/j.oooo.2021.08.015 34593340 PMC9112430

[22] Wolff A Joshi RK Ekstrom J Aframian D Pedersen AML Proctor G A guide to medications inducing salivary gland dysfunction, Xerostomia, and subjective sialorrhea: a systematic review sponsored by the world workshop on oral medicine Vi. Drugs RD. (2017) 17:1–28. 10.1007/s40268-016-0153-9 PMC5318321 27853957

[23] Aliko A Wolff A Dawes C Aframian D Proctor G Ekström J World workshop on oral medicine vi: clinical implications of medication-induced salivary gland dysfunction. Oral Surg Oral Med Oral Pathol Oral Radiol. (2015) 120:185–206. 10.1016/j.ooo.2014.10.027 25861957

[24] Miranda-Rius J Brunet-Llobet L Lahor-Soler E Farré M. Salivary secretory disorders, inducing drugs, and clinical management. Int J Med Sci. (2015) 12:811–24. 10.7150/ijms.12912 26516310 PMC4615242

[25] Screebny LM Valdini A. Xerostomia. A negnected symptom. Arch Intern Med. (1987) 147:1333–7. 10.1001/archinte.147.7.1333 3300589

[26] Guggenheimer J Moore PA. Xerostomia: etiology, recognition and treatment. J Am Dent Assoc. (2003) 134:61–9. 10.14219/jada.archive.2003.0018 12555958

[27] Locker D. Subjective reports of oral dryness in an older adult population. Comm Dent Oral Epidem. (1993) 21:165–8. 10.1111/j.1600-0528.1993.tb00744.x 8348792

[28] Rossie K Guggenheimer J. Oral candidiasis: clinical manifestations, diagnosis and treatment. Pract Periodontics Aesthet Dent. (1997) 9:635–42. PMID: . 9573835

[29] Lee YC Hong IK Na SY Eun YG. Evaluation of salivary function in patients with burning mouth syndrome. Oral Dis. (2015) 21:308–13. 10.1111/odi.12270 24962264

[30] Pajukoski H Meurman JH Halonen P Sulkava R. Prevalence of subjective dry mouth and burning mouth in hospitalized elderly patients and outpatients in relation to Saliva, medication, and systemic diseases. Oral Surg Oral Med Oral Pathol Oral Radiol Endod. (2001) 92:641–9. 10.1067/moe.2001.118478 11740482

[31] Nagler RM Hershkovich O. Sialochemical and gustatory analysis in patients with oral sensory complaints. J Pain. (2004) 5:56–63. 10.1016/j.jpain.2003.09.002 14975379

[32] Pekiner FN Gümrü B Demirel GY Ozbayrak S. Burning mouth syndrome and Saliva: detection of salivary trace elements and cytokines. J Oral Pathol Med. (2009) 38:269–75. 10.1111/j.1600-0714.2008.00734.x 19141055

[33] Soares MS Chimenos-Küstner E Subirá-Pifarrè C Rodríguez de Rivera-Campillo ME López-López J. Association of burning mouth syndrome with Xerostomia and medicines. Med Oral Patol Oral Cir Bucal. (2005) 10:301–8. PMID: . 16056186

[34] Suh KI Lee JY Chung JW Kim YK Kho HS. Relationship between salivary flow rate and clinical symptoms and behaviours in patients with dry mouth. J Oral Rehabil. (2007) 34:739–44. 10.1111/j.1365-2842.2006.01712.x 17824886

[35] Toida M Nanya Y Takeda-Kawaguchi T Baba S Iida K Kato K Oral complaints and stimulated salivary flow rate in 1188 adults. J Oral Pathol Med. (2010) 39:407–19. 10.1111/j.1600-0714.2009.00852.x 20202092

[36] Glazar I Urek MM Brumini G Pezelj-Ribaric S. Oral sensorial complaints, salivary flow rate and mucosal lesions in the institutionalized elderly. J Oral Rehabil. (2010) 37:93–9. 10.1111/j.1365-2842.2009.02027.x 19968768

[37] Acharya S Hägglin C Jontell M Wenneberg B Ekström J Carlén A. Saliva on the oral Mucosa and whole Saliva in women diagnosed with burning mouth syndrome. Oral Dis. (2018) 24:1468–76. 10.1111/odi.12918 29917294

[38] Hershkovich O Nagler RM. Biochemical analysis of Saliva and taste acuity evaluation in patients with burning mouth syndrome, Xerostomia and/or gustatory disturbances. Arch Oral Biol. (2004) 49:515–22. 10.1016/j.archoralbio.2004.01.012 15126133

[39] Poon R Su N Ching V Darling M Grushka M. Reduction in unstimulated salivary flow rate in burning mouth syndrome. Br Dent J. (2014) 217:E14. 10.1038/sj.bdj.2014.884 25303607

[40] Spadari F Venesia P Azzi L Veronesi G Costantino D Croveri F Low basal salivary flow and burning mouth syndrome: new evidence in this enigmatic pathology. J Oral Pathol Med. (2015) 44:229–33. 10.1111/jop.12240 25155153

[41] Rouleau TS Shychuk AJ Kayastha J Lockhart PB Nussbaum ML Brennan MT. A retrospective, cohort study of the prevalence and risk factors of oral burning in patients with dry mouth. Oral Surg Oral Med Oral Pathol Oral Radiol Endod. (2011) 111:720–5. 10.1016/j.tripleo.2011.01.042 21497523

[42] Ko JY Kim MJ Lee SG Kho HS. Outcome predictors affecting the efficacy of clonazepam therapy for the management of burning mouth syndrome (bms). Arch Gerontol Geriatr. (2012) 55:755–61. 10.1016/j.archger.2011.10.001 22040716

[43] de Souza FT Amaral TM dos Santos TP Abdo EN Aguiar MC Teixeira AL Burning mouth syndrome: a therapeutic approach involving mechanical salivary stimulation. Headache. (2012) 52:1026–34. 10.1111/j.1526-4610.2011.02037.x 22084903

[44] Dawes C. Summary of: reduction in unstimulated salivary flow rate in burning mouth syndrome. Br Dent J. (2014) 217:364–5. 10.1038/sj.bdj.2014.885 25303588

[45] Iorgolescu G. Saliva between normal and pathological. Important factors in determining systemic and oral health. J Med Life. (2009) 2:303–7. PMID: . 20112475 PMC5052503

[46] Kao CH HY ChangLai SP Liao KK. Evidence for decreased salivary function in patients with reflux esophagitis. Digestion. (1999) 60:191–5. 10.1007/s00455-005-0016-y 10343131

[47] Koshiyama S Tanimura K Ito K Funayama S Hira D Komase Y Gastroesophageal reflux-like symptoms are associated with hyposalivation and oropharyngeal problems in patients with asthma. Resp Invest. (2021) 59:114–9. 10.1016/j.resinv.2020.06.004 32665193

[48] Watanabe M Nakatani E Yoshikawa H Kanno T Nariai Y Yoshino A Oral soft tissue disorders are associated with gastroesophageal reflux disease: retrospective study. BMC Gastroenterol. (2017) 17:92. 10.1186/s12876-017-0650-5 28784097 PMC5545853

[49] Yoshikawa H Furuta K Ueno M Egawa M Yoshino A Kondo S Oral symptoms including dental erosion in gastroesophageal reflux disease are associated with decreased salivary flow volume and swallowing function. J Gastroenterol. (2012) 47:412–20. 10.1007/s00535-011-0515-6 22200941

[50] Lechien JE Hans S De Marrez LG Deuqanter D Rodriguez A Muls V Prevalence and features of laryngopharyngeal reflux in patients with primary burning mouth syndrome. Laryngoscope. (2021) 131:1–7. 10.1002/lary.29604 34009647

[51] Hakeem A Fitzpatrick SG Bhattacharyya I Islam MN Cohen DM. Clinical characterization and treatment outcome of patients with burning mouth syndrome. Gen Dent. (2018) 66:41–7. PMID: . 29714699

[52] Sánchez-Blanco I Rodriguez-Téllez M Corcuera-Flores JR González-Blanco C Torres-Lagares D Serrera-Figallo MÁ Effectiveness of salivary stimulation using Xylitol-malic acid tablets as coadjuvant treatment in patients with gastro-oesophageal reflux disease: early findings. Med Oral Patol Oral Cir Bucal. (2020) 25:e818–26. 10.4317/medoral.23887 33037808 PMC7648928

[53] Di Fede O Di Liberto C Occhipinti G Vigneri S Lo Russo L Fedele S Oral manifestations in patients with gastro-oesophageal reflux disease: a single-center case-control study. J Oral Pathol Med. (2008) 37:336–40. 10.1111/j.1600-0714.2008.00646.x 18284539

[54] Campisi G Lo Russo L Di Liberto C Di Nicola F Butera D Vigneri S Saliva variations in gastro-oesophageal reflux disease. J Dent. (2008) 36:268–71. 10.1016/j.jdent.2008.01.003 18313197

[55] Correa MC Lerco MM Henry MA. Study in oral cavity alterations in patients with gastroesophageal reflux disease. Arq Gastroenterol. (2008) 45:132–6. 10.1590/S0004-28032008000200008 18622467

[56] Ristevska I Armata RS D’Ambrosio C Furtado M Anand L Katzman MA. Xerostomia: understanding the diagnosis and the treatment of dry mouth. J Fam Med Dise Prev. (2015) 1:2.

[57] Gil-Montoya JA Silvestre FJ Barrios R Silvestre-Rangi J. Treatment of Xerostomia and hyposalivation in the elderly: a systematic review. Med Oral Patol Oral Cir Bucal. (2016) 21:e355–66. 10.4317/medoral.20969 27031061 PMC4867210

[58] Lopez-Pintor RM Casanas E González-Serrano J Serrano J Ramirez L de Arriba L Xerosotmia, hyposalivation, and salivary flow in diabetes patients. J Diabetes Res. (2016) 2016:4372852. 10.1155/2016/4372852 27478847 PMC4958434

[59] Thakkar JP Lane C. Hyposalivation and Xerostomia and burning mouth syndrome. Medical management. Oral Maxillofac Surg Clin N Am. (2022) 34:135–46. 10.1016/j.coms.2021.08.002 34598858

[60] Thomson WM van der Putten GJ de Baat C Ikebe K Matsuda K Enoki K Shortening the Xerostomia inventory. Oral Surg Oral Med Oral Pathol Oral Radiol Endod. (2011) 112:322–27. 10.1016/j.tripleo.2011.03.024 21684773 PMC3154566

[61] Navazesh M Christensen CM. A comparison of whole mouth resting and stimulated salivary measurement procedures. J Dent Res. (1982) 61:1158–62. 10.1177/00220345820610100901 6956596

[62] Anderson DJ Hector MP. Periodontal mechanoreceptors and parotid secretion in animals and man. J Dent Res. (1987) 66:518–23. 10.1177/00220345870660022201 3476568

[63] Dawes C. Physiological factors affecting salivary flow rate, oral sugar clearance, and the sensation of dry mouth in man. J Dent Res. (1987) 66:648–53. 10.1177/00220345870660S107 3476629

[64] Orellana MF Lagravére MO Boychuk DGJ Major PW Flores-Mir C. Prevalence of Xerostomia in population-based samples: a systematic review. Publ Health Dent. (2006) 66:152–8. 10.1111/j.1752-7325.2006.tb02572.x 16711637

[65] Diep MT Jensen JL Skudutyte-Rysstad R Young A Sødal ATT Petrovski BÉ Xerostomia and hyposalivation among a 65-yr-old population living in Oslo, Norway. Eur J Oral Sci. (2021) 129:e12757. 10.1111/eos.12757 33501713 PMC7986810

[66] Wiener CR Wu B Crout R Wiener M Plassman B Kao E Hyposalivation and Xerostomia in dentate older adults. J Am Dent Assoc. (2010) 141:279–84. 10.14219/jada.archive.2010.0161 20194383 PMC2899485

[67] Jamieson LM Thomson WM. Xerostomia: its prevalence and associations in the adult Australian population. Aust Dent J. (2020) 65(Suppl 1):S67–70. 10.1111/adj.12767 32583587

[68] Imura H Shimada M Yamazaki Y Sugimoto K. Characteristic changes of Saliva and taste in burning mouth syndrome patients. J Oral Pathol Med. (2016) 45(3):231–6. 10.1111/jop.12350 26293497

[69] Ericsson Y Hardwick L. Individual diagnosis, prognosis and counselling for caries prevention. Caries Res. (1978) 12:94–102. 10.1159/000260369 277298

[70] Bardow A Nyvad B Nauntofte B. Relationships between medication intake, complaints of dry mouth, salivary flow rate and composition, and the rate of tooth demineralization in situ. Arch Oral Biol. (2001) 46:413–23. 10.1016/s003-9969(01)00003-6 11286806

[71] Navazesh M Brightman VJ Pogoda JM. Relationship of medical Status, medications, and salivary flow rates in adults of different ages. Oral Surg Oral Med Oral Pathol Oral Radiol Endod. (1996) 81:172–6. 10.1016/s1079-2104(96)80410-0 8665310

[72] Alwaheidi HAA O’Toole S Bernabé E. The interrelationship between Xerogenic medication use, subjective oral dryness and tooth wear. J Dent. (2021) 104:103542. 10.1016/j.jdent.2020.103542 33276080

[73] Leal SC Bittar J Portugal A Falcao DP Faber J Zanotta P. Medication in elderly people: its influence on salivary pattern, signs and symptoms of dry mouth. Gerodontology. (2010) 27:129–33. 10.1111/j.1741-2358.2009.00293.x 20337727

[74] Krunic J Stojanovic N Ivkovic N Stojic D. Salivary flow rate and decayed, missing, and filled teeth (dmft) in female patients with schizophrenia on chlorpromazine therapy. J Dent Sci. (2013) 8:418–24. 10.1016/j.jds.2013.05.004

[75] Narhi TO Meurman JH Ainamo A Nevalainen JM Schmidt-Kaunisaho KG Siukosaari P Association between salivary flow rate and the use of systemic medication among 76-, 81-, and 86-year-old inhabitants in Helsinki, Finland. J Dent Res. (1992) 71:1875. 10.1177/00220345920710120401 1452886

[76] Wu AJ Ship JA. A characterization of Major salivary gland flow rates in the presenceof medications and systemic diseases. Oral Surg Oral Med Oral Pathol. (1993) 76:301–6. 10.1016/0030-4220(93)90258-6 8378045

[77] Meurman JH Rantonen P. Salivary flow rate, buffering capacity, and yeast counts in 187 consecutive adult patients from kuopio, Finland. Scand J Dent Res. (1994) 102:229–34. 10.1111/j.1600-0722.1994.tb01185.x 8091123

[78] Djukic L Roganovic J Brajovic MD Bokonjic D Stojic D. The effects of antihypertensives and type 2 diabetes on salivary flow and total antioxidant capacity. Oral Dis. (2015) 21:619–25. 10.1111/odi.12325 25689395

[79] Korsten MA Rosman AS Fishbein S Shlein RD Goldberg HE. Chronic Xerostomia increases esophageal acid exposure and is associated with esophageal injury. Am J Med. (1991) 90:701–6. 10.1016/0002-9343(91)90665-K 2042685

[80] Sonnenberg A Steinkamp U Weise A Berges W Wienbeck M Rohner HG Salivary secretion in reflux esophagitis. Gastroenterol. (1982) 83:889–95. 10.1016/S0016-5085(82)80021-8 7106518

[81] Helm JF Dodds W Hogan WJ. Salivary response to esophageal acid in normal subjects and patients with reflux esophagitis. Gastroenterol. (1987) 93:1393–7. 10.1016/0016-5085(87)90270-8 3678754

[82] Namiot Z Rourk RM Piascik R Hetzel DP Sarosiek J McCallum RW. Interrelationship between esophageal challenge with mechanical and chemical stimuli and salivary protective mechanisms. Am J Gastroenterol. (1994) 89:581–7 PMID: . 8147362

[83] Costa HO Neto OM Eckley CA. Is there a relationship between the ph and volume of Saliva and esophageal ph-metry results? Dysphagia. (2005) 20:175–81. 10.1007/s00455-005-0016-y 16362506

[84] Sarosiek J Scheurich CJ Marcinkiewicz M McCallum RW. Enhancement of salivary esophagoprotection: rationale for a physiological approach to gastroesophageal reflux disease. Gastroenter. (1996) 110:675–81. 10.1053/gast.1996.v110.pm8608875 8608875

[85] Navazesh M Christensen CM Brightman C. Clinical criteria for the diagnosis of salivary gland hypofunction. J Dent Res. (1992) 71:1363–9. 10.1177/00220345920710070301 1629451

[86] Hunter KD Wilson WS. The effects of antidepressant drugs on salivary flow and content of sodium and potassium ions in human parotid Saliva. Archs Oral Biol. (1995) 40:983–89. 10.1016/0003-9969(95)00079-5 8670028

[87] van Hooff M van Baak M Schols M Rahn KH. Studies of salivary flow in borderline hypertension: effects of drugs acting on structures innervated by the autonomic nervous system. Clin Sci (Lond). (1984) 66:599–604. 10.1042/cs0660599 6705484

